# Scalable Synthesis of Thermally Robust Carbon Dot-Silica Microspheres Enabling High-Temperature Polymer Processing and Multifunctional Luminescent Composites

**DOI:** 10.1021/acsami.5c22439

**Published:** 2026-01-08

**Authors:** Hirohisa Iwabayashi, Kenji Okada, Arisa Fukatsu, Ryohei Mori, Masahide Takahashi

**Affiliations:** a Department of Materials Science, Graduate School of Engineering, 12936 Osaka Metropolitan University, Osaka 599-8531, Japan; b Research Division, Fuji Pigment Co., Hyogo 666-0015, Japan

**Keywords:** carbon dots, silica encapsulation, thermal stability, fluorescent composites, injection molding, optical properties, spray drying

## Abstract

Carbon dots (C-dots) are promising fluorescent nanomaterials, yet their inherent thermal and environmental instability, coupled with challenges in achieving independent control over emission wavelength, intensity, and composite transparency in polymer matrices, severely limit their practical utility. Here, we present a scalable spray-drying strategy to synthesize C-dot-embedded silica (SiO_2_) microspheres featuring a dense shell with low accessible porosity. This unique, dense SiO_2_ shell effectively encapsulates C-dots, providing remarkable thermal stability up to 350 °C. Our method also enables precise tuning of emission wavelength from blue to yellow by simply adjusting the C-dot concentration within the spheres during synthesis. Crucially, the incorporation of these C-dot-embedded SiO_2_ microspheres into PMMA matrices allows for independent control of composite transparency and fluorescence intensity by varying the microsphere loading, all without altering the emission wavelength. Demonstrating their unprecedented industrial applicability, these robust microspheres are fully compatible with high-temperature injection molding processes (up to 250 °C), previously unsuitable for C-dots, enabling the formation of complex 3D fluorescent objects. Furthermore, this approach facilitates a “hidden-to-revealed” functionality, where an embedded fluorescent object remains invisible under ambient light but brightly emerges upon UV illumination, offering vast potential for advanced security features, interactive displays, and innovative consumer products. This work significantly broadens the applicability of inherently sensitive C-dots to widely utilized industrial polymer processing techniques, paving the way for their large-scale integration into high-performance, versatile luminescent materials.

## Graphical abstract

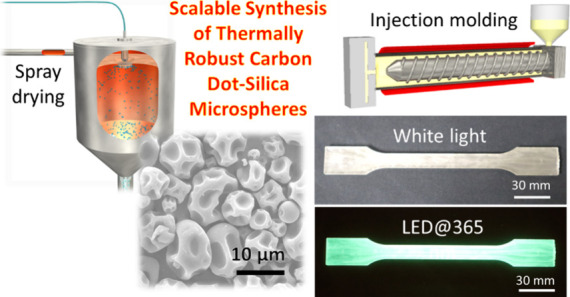

## 1 Introduction

Carbon dots (C-dots), a class of fluorescent nanomaterials, have garnered significant attention in diverse fields such as display technology and bioimaging due to their unique optical properties and biocompatibility.^1–4^ Embedding C-dots into polymer matrices holds promise for developing novel functional materials, such as highly efficient light-emitting devices, advanced sensors, and next-generation bioimaging probes. However, the inherent limitations of conventional C-dots, including their poor thermal and environmental stability, have hindered the achievement of long-term stability and uniform dispersion within resin systems. Notably, attempts to control the emission intensity and transparency of composite materials by adjusting the C-dot content often lead to aggregation and undesirable shifts in their fluorescence wavelength, significantly restricting their application potential.

To address the stability issues of C-dots, various encapsulation strategies using materials like polymers and silica have been explored.^5–8^ While polymeric encapsulation offers advantages in flexibility and processability, it often falls short in providing sufficient thermal stability and long-term barrier properties against environmental degradation, especially for high-temperature applications. In contrast, silica particles are recognized for their superior thermal stability, excellent chemical inertness, and ability to form robust matrices, making them a more desirable choice for protecting C-dots under harsh conditions and ensuring uniform dispersion within various resin systems. Nevertheless, silica particles produced via conventional sol–gel methods, such as the Stöber process, often exhibit a porous structure.^9–13^ These pores fail to provide a complete barrier against the infiltration of moisture and oxygen from the external environment, consequently leading to the degradation of the C-dots’ fluorescence properties. Therefore, the development of an effective method for the facile and uniform dispersion of C-dots in general-purpose resins, while preserving their desirable characteristics, remains a critical challenge.

In this study, we addressed the aforementioned limitations by employing a scalable spray-drying technique^14–18^ with a conventional sol–gel solution to synthesize silica (SiO_2_) microspheres encapsulating C-dots with high surface compactness. Strikingly, the resulting C-dot-embedded SiO_2_ microspheres exhibited a lower specific surface area and higher density compared to silica particles prepared by conventional sol–gel methods including the Stöber process. This unique structure effectively shielded the C-dots from the external environment, particularly preventing oxidation at elevated temperatures. Consequently, the C-dots retained excellent thermal stability, enabling their uniform dispersion within poly­(methyl methacrylate) (PMMA), a widely used general-purpose resin. Furthermore, by precisely controlling the C-dot concentration within the silica spheres, we achieved fine-tuning of the emission wavelength of the composite material. Moreover, the emission intensity and transparency of the composite could be independently controlled by adjusting the concentration of the C-dot-embedded silica spheres within the PMMA matrix, unlocking unprecedented functionalities. This approach also holds industrial advantages for applications in large-area displays, flexible devices, and biocompatible medical devices.^19–26^

## 2 Experimental Section

### 2.1 Materials

Tetraethyl orthosilicate (TEOS, > 98.0% (GC)) and ammonia (NH_3_, 28–30%) were procured from Tokyo Chemical Industry Co., Ltd. (TCI). Ethanol (99.5%) and nitric acid (HNO_3_, specific gravity approximately 1.38) were obtained from Fujifilm Wako Pure Chemical Corporation. Carbon dots (C-dots), specifically Fuji GQD 818, were procured from Fuji Pigment Co., Ltd. and utilized without further purification. The concentrated nitric acid was diluted to 1 M with deionized water prior to use. PMMA (ACRYPET VH001) was obtained from Mitsubishi Chemical Corporation (Tokyo, Japan) and used as received.

### 2.2 Methods

#### 2.2.1 Preparation of C-Dot-Embedded SiO_2_ Microspheres

In this study, we employed a spray-drying method to synthesize C-dot-embedded silica (SiO_2_) microspheres with high surface compactness. Spray drying is a widely utilized technique for preparing inorganic microspheres from sol–gel solutions due to its scalability and ability to control particle morphology.^27–29^ This process involves atomizing liquid precursors into fine droplets, which are then rapidly dried by contact with a hot drying medium, yielding powdered products. Specifically, we prepared a C-dot-dispersed silica sol–gel precursor solution (Figure 1a,b). The precursor sol was prepared by sequentially mixing tetraethyl orthosilicate (TEOS, 350 mL, 1.49 mol, accounting for ∼95% purity), ethanol (45 mL, 0.77 mol, ∼99.5% purity), deionized water (40 mL, 2.22 mol) containing a predetermined concentration of C-dots, and 1 M HNO_3_ aqueous solution (30 mL). The mixture was stirred thoroughly in a 500 mL lidded polypropylene (PP) container. In the calculation of the molar ratios, the water content within the 1 M HNO_3_ aqueous solution (approximately 6 wt %) was taken into account, resulting in a total water content of 3.83 mol. The effective water-to-TEOS molar ratio (R = [H_2_O]/[Si]) was 2.57, and the acid-to-silicon molar ratio ([H^+^]/[Si]) was 0.020. The resulting sol was aged for 24 h at room temperature prior to spray drying. The pH of the sol remained constant at approximately 3 throughout the aging period, providing a stable environment for the encapsulated C-dots. At the time of spray drying, the sol remained a stable, transparent liquid with low viscosity, exhibiting no gelation or precipitation. Subsequently, the prepared solution was continuously fed by a pump to a rotating disk atomizer (18,000 rpm) within a spray dryer. The atomized fine droplets were rapidly dried by contact with hot air in a chamber maintained at 80 °C, leading to the formation of powdered C-dot-embedded SiO_2_ microspheres (Figure 1c). The feeding was performed without continuous stirring or prefiltration. No significant clogging of the atomization nozzle was observed during the process, confirming the stability of the feed solution. The resulting powder was collected from the bottom outlet of the drying chamber. For purification, the obtained microspheres were meticulously washed with deionized water and ethanol using an ultrasonic cleaner, followed by drying in an oven at 60 °C for 2 h. The absence of C-dot elution after washing was confirmed by analyzing the fluorescence spectrum of the washing solution.

**Figure 1:**
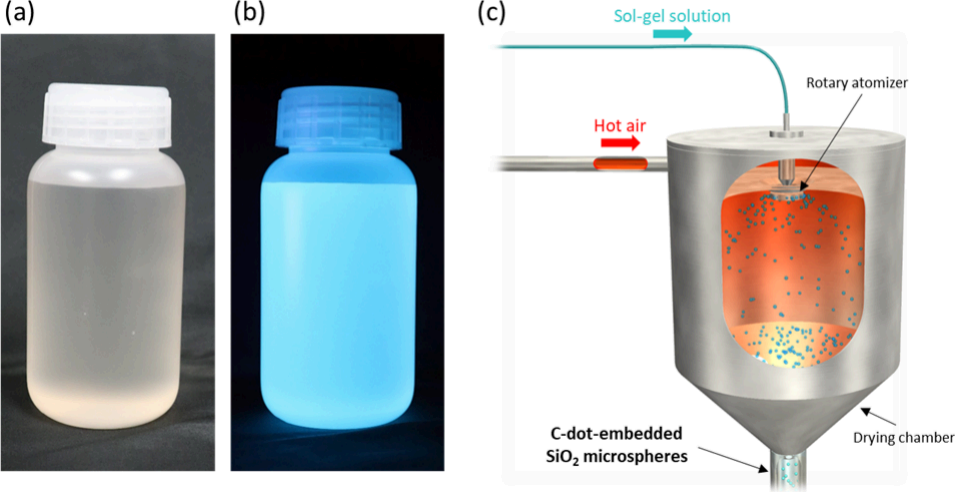
Photos of C-dot-dispersed silica sol–gel precursor solution under white light (a) and UV light (405 nm) (b). (c) A schematic illustration showing spray drying process using sol–gel solution.

#### 2.2.2 Preparation of Comparative Silica Samples

Silica microspheres by Stöber method: Comparative silica microspheres were synthesized following a modified Stöber method.^30^ Briefly, 4.5 mol of deionized water, 0.6 mol of ammonia (NH_3_), and 0.34 mol of ethanol were mixed in an Erlenmeyer flask under stirring. A solution containing 0.05 mol of TEOS and 1.4 mol of ethanol was then added dropwise from a buret. The reaction mixture was stirred for 1 h. The precipitated product was collected by centrifugation, washed sequentially with deionized water and ethanol, and subsequently dried overnight to obtain the silica microspheres.

Bulk silica: Bulk silica was prepared according to the method described in a previous study.^31^ The obtained bulk material was then pulverized into a fine powder using an alumina mortar for characterization.

#### 2.2.3 Preparation of PMMA/C-Dot-Embedded SiO_2_ Composites and Injection Molding

To prepare the fluorescent composites, PMMA (ACRYPET VH001, Mitsubishi Chemical Corporation) was used as the polymer matrix. First, the C-dot-embedded SiO_2_ microspheres were melt-compounded with PMMA to produce the masterbatches using a twin-screw extruder (KZW15TW, Technovel Corporation). The extrusion was performed at a heater temperature of 250 °C, and the material was passed through the extruder three times to ensure the uniform dispersion of the microspheres within the resin. Subsequently, the resulting masterbatches were processed into the final specimens using a benchtop injection molding machine (Mold Lock, Century Innovation Co., Ltd.) equipped with a standard dumbbell-shaped mold. The injection molding was conducted at a melt temperature of 250 °C and a mold temperature of 140 °C.

### 2.3 Characterization

The dispersion of C-dots within the SiO_2_ microspheres was investigated using a scanning transmission electron microscope (STEM, JEOL JEM-2100Plus) equipped with an energy dispersive X-ray spectrometer (EDS). For STEM observations, the SiO_2_ microspheres were mechanically crushed and dispersed in ethanol via sonication. The supernatant of the resulting dispersion was then cast onto a copper TEM grid. The morphology of the C-dot-embedded SiO_2_ microspheres was observed using a scanning electron microscope (SEM, Hitachi FlexSEM 1000II). X-ray diffraction (XRD) using a Rigaku SmartLab diffractometer was used to confirm the formation of amorphous silica. Fluorescence spectra were acquired using a Jasco FP-8300 fluorescence spectrophotometer. Optical transmittance was measured with an Ocean Photonics OPFLUX-020 total luminous flux measurement system, utilizing a 1 mW/530 nm laser diode as the light source. Photoluminescence intensity at 520 nm upon 450 nm excitation was measured using a Shimadzu RF-5300pc fluorescence spectrophotometer. For the thermal stability evaluation, 100 mg of C-dot-embedded SiO_2_ microspheres (C-dots: 0.6 wt %) were placed in 1.5 mL glass vials and heated in a muffle furnace at preset temperatures ranging from 50 to 350 °C for 1 h. After cooling to room temperature, fluorescence spectra were acquired using the fluorescence spectrophotometer. Photoluminescence intensity at 520 nm upon 450 nm excitation was measured using the fluorescence spectrophotometer. Particle size distribution was determined by dynamic image analytics (DIA) using a KEYENCE VHX Digital Microscope, which was chosen over laser diffraction (LD) to mitigate influences from surface roughness. Specific surface area and true density of the C-dot-embedded SiO_2_ microspheres were measured using a MICROTRAC BELSORP MAX II Specific Surface Area Analyzer. True density measurements employed helium (He) as the displacement gas, consistent with established protocols. Prior to the measurements, the samples were degassed at 120 °C under vacuum for 6 h. Photographs of sample luminescence were captured using colored glass filters to suppress the excitation light: an SCF-50S-44Y (SIGMAKOKI) filter was used for 365 nm excitation, and an SCF-50S-48Y (SIGMAKOKI) filter was used for 405 nm excitation. Magnified photos of the silica-composite masterbatch and injection-molded products were obtained using a KEYENCE VHX Digital Microscope.

## 3 Results and Discussion

### 3.1 Synthesis and Characterization of C-Dot-Embedded SiO_2_ Microspheres

A scanning electron microscopy (SEM) image of the synthesized C-dot-embedded SiO_2_ microspheres, prepared with 6.0 × 10^–1^ wt % C-dot in the sol–gel solution, is presented in Figure 2a. Regardless of the C-dot concentration, we consistently observed the formation of ″dimpled″ silica microspheres, characterized by distinctive indentations on their surface (Figures S1 and S2). These microspheres primarily ranged in diameter from 5 to 7 μm, with an overall distribution from 1 to 15 μm (Figures S1 and S2 and Table S1). The formation mechanism of these dimpled silica microspheres will be discussed in the following section. Furthermore, scanning transmission electron microscopy-energy dispersive X-ray spectroscopy (STEM-EDS) analysis confirmed the uniform dispersion of C-dots throughout the SiO_2_ microspheres, indicating successful encapsulation of C-dots within the silica matrix (Figure 2b–d and Figure S3). The N_2_ adsorption–desorption isotherms of the C-dot-embedded SiO_2_ microspheres are presented in Figure 3. For comparison, bulk silica prepared by conventional sol–gel methods and silica microspheres synthesized via the Stöber method are known to exhibit significant nitrogen uptake and pronounced hysteresis loops due to the presence of micro- and mesopores formed during gelation and solvent drying, as reported in previous studies.^31^ In stark contrast, no discernible gas adsorption was observed in the present C-dot-embedded SiO_2_ microspheres synthesized by the spray-drying method. This absence of adsorption indicates the formation of a shell with low accessible porosity that effectively prevents gas diffusion into the interior of the SiO_2_ microspheres. Indeed, the specific surface areas determined by the BET method were below the limit of quantitation (LOQ, < 1 m^2^/g), 61 m^2^/g and 170 m^2^/g for C-dot-embedded SiO_2_ microspheres and silica bulk prepared by a conventional sol–gel method and silica microspheres by the Stöber method, respectively. Moreover, SEM observation of the cross sections of the fractured C-dot-embedded SiO_2_ microspheres revealed no apparent voids, further confirming their compact structure (Figure S4).

**Figure 2:**
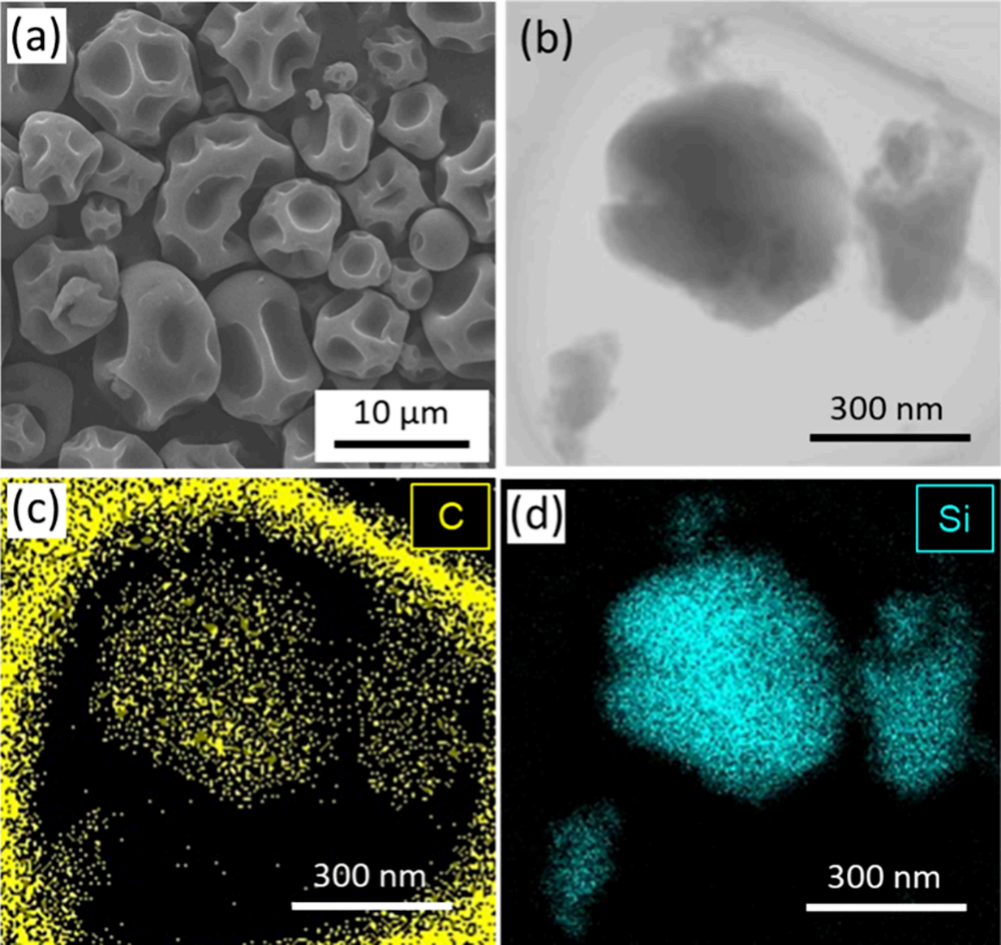
(a) A SEM image of C-dot-embedded SiO_2_ microspheres (6.0 × 10^–1^ wt % C-dot). (b-d) STEM image and EDS elemental maps of crushed fragments of the C-dot-embedded SiO_2_ microsphere; (b) high-angle annular dark-field (HAADF) image, (c) EDS mapping image for carbon, (d) EDS mapping image for silicon, showing the distribution of C-dots within the microsphere. Note that the carbon signal is also detected from the carbon grid supporting the sample.

**Figure 3:**
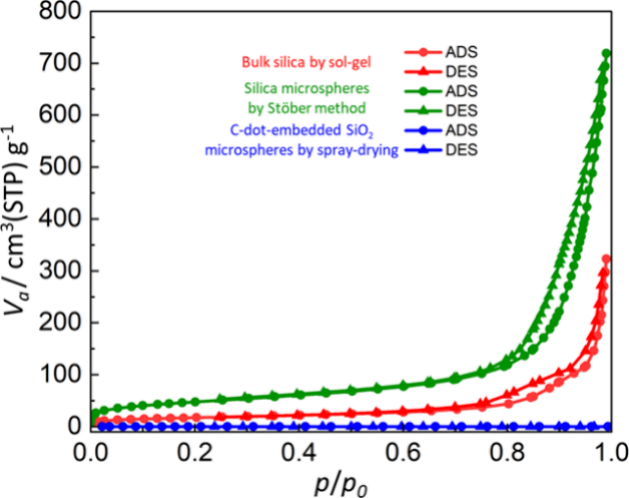
N_2_ isotherm for C-dot-embedded SiO_2_ microspheres, crushed silica powder from its bulk prepared by a sol–gel method, and silica prepared by Stöber method.

Furthermore, density of the C-dot-embedded SiO_2_ microspheres, derived from gas adsorption data, yielded a value of 1.9 g/cm^3^. This value is slightly lower than that of commercially available pure silica (2.1 g/cm^3^) measured by the same method, which suggests the presence of approximately 10% inaccessible micropores within the SiO_2_ microspheres. Considering the N_2_ gas adsorption–desorption results, this indicates that a highly dense structure is predominantly formed on the particle surface, preventing N_2_ gas from accessing these internal micropores. The formation of this dense, nonporous surface structure is attributed to the rapid solvent (water and ethanol) evaporation at the droplet surface during spray drying, which leads to initial gelation and the rapid formation of a rigid SiO_2_ shell. Subsequent solvent evaporation and gelation within the sphere’s interior cause volume shrinkage, further densifying the preformed surface rigid SiO_2_ layer. This rapid surface drying and gelation create a temporarily hard surface and a softer interior. The subsequent internal volume shrinkage then induces in-plane compressive stress on the surface SiO_2_ layer, leading to the formation of the observed dimple structure. Such dimple or wrinkle structures, arising from mechanical instability between the surface and interior, are commonly observed in sol–gel derived films and spheres.^32–36^ The formation of such a highly dense SiO_2_ surface layer, impenetrable to gas, significantly enhances the retention of C-dots within the C-dot-embedded SiO_2_ microspheres. For instance, no elution of C-dots into the solution was observed after C-dot-embedded SiO_2_ microspheres were dispersed in deionized water, stirred, and then allowed to stand for 1 week (Figure S5).

### 3.2 Optical Properties of C-Dot-Embedded SiO_2_ Microspheres

All C-dot-embedded SiO_2_ microspheres synthesized from silica sol–gel precursor solution with five different C-dot concentrations ranging from 3.0 × 10 ^–2^ wt % to 6.0 × 10 ^–1^ wt % exhibited strong photoluminescence (Figure 4). The excitation–emission intensity spectra of these C-dot-embedded SiO_2_ microspheres are presented in Figure 4b–d. As the C-dot concentration increased, we observed a significant red-shift in the emission maximum wavelength from 445 to 520 nm. This can be attributed to the shorter relative distance between the C-dots at higher concentrations, as similarly explained in previous studies.^31,37^ It is important to note that the fluorescence properties of these C-dots, once encapsulated within the silica microspheres, remained stable without any noticeable change even after approximately five years, highlighting the excellent long-term stability conferred by the silica encapsulation.

**Figure 4:**
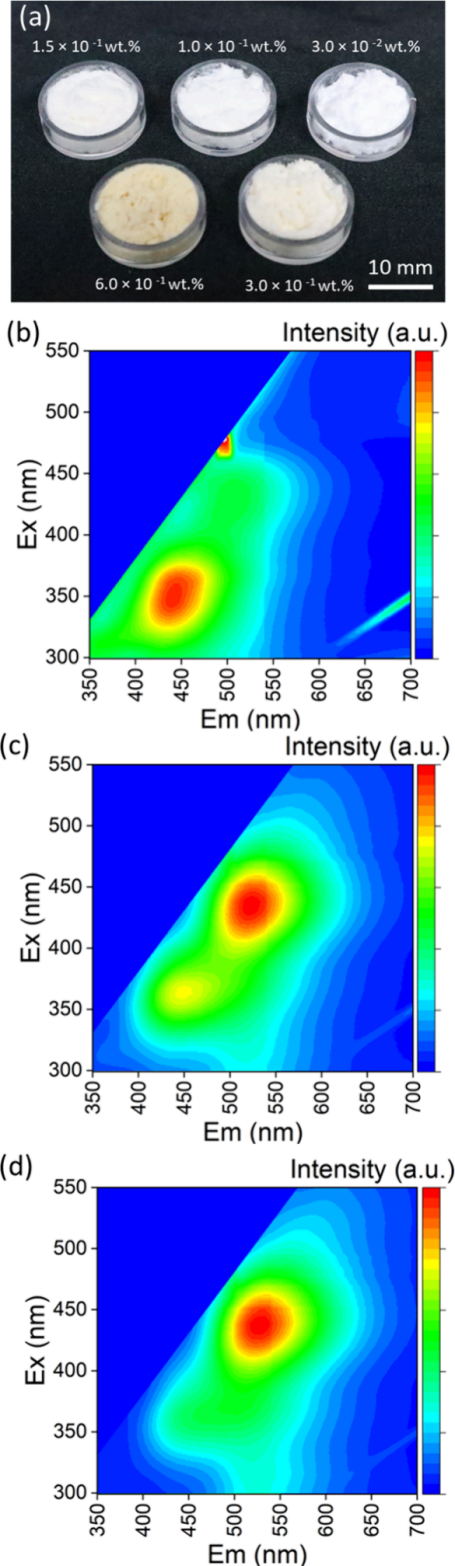
(a) Photos of C-dot-embedded SiO_2_ microspheres synthesized from silica sol–gel precursor solution with five different C-dot concentrations ranging from 3.0 × 10 ^–2^ wt % to 6.0 × 10 ^–1^ wt % under white light. Excitation–emission intensity spectra of the C-dot-embedded SiO_2_ microspheres with different C-dot concentrations; (b) 3.0 × 10 ^–2^ wt %, (c) 3.0 × 10 ^–1^ wt %, (d) 6.0 × 10 ^–1^ wt %.

### 3.3 Versatile Processing and Enhanced Thermal Stability for Practical Applications

The C-dot-embedded SiO_2_ microspheres synthesized in this study are fine powders, which can be readily incorporated into polymer or other inorganic matrices to form fluorescent films or three-dimensional (3D) fluorescent bodies. This capability holds promise for enabling white-light LEDs by simply combining them with blue LED chips in a variety of applications. To demonstrate this versatile applicability, we blended C-dot-embedded SiO_2_ microspheres (6.0 × 10^–1^ wt % C-dot) into poly­(methyl methacrylate) (PMMA) resin, a common matrix material for phosphors, to fabricate fluorescent disks (Figure S6). We prepared these disks with varying concentrations of C-dot-embedded SiO_2_ microspheres in the PMMA resin (1, 3, 5, and 10 wt %) and evaluated the relationship between their optical transmittance and fluorescence properties (Figure 5). We found that a lower concentration of C-dot-embedded SiO_2_ microspheres in the PMMA resin resulted in higher transmittance, while a higher concentration led to increased fluorescence intensity. Notably, the fluorescence wavelength remained unchanged across all these disks (Figure S7). Since the fluorescence wavelength of C-dots depends on the relative distance between C-dots, it is primarily controlled by the C-dot concentration in the precursor sol–gel solution. Therefore, by adjusting the proportion of C-dot-embedded SiO_2_ microspheres in the PMMA resin, we can control the optical transmittance and fluorescence intensity, without altering the fluorescence wavelength, tailoring them for specific applications. This represents a significant advantage; previously, while C-dot-embedded polymer composite phosphors have been reported, their transparency/fluorescence intensity has typically been controlled by varying the C-dot concentration within the matrix.^38^ This approach often leads to changes in the inter-C-dot distance, consequently shifting the fluorescence wavelength and also causing concentration quenching, making it challenging to independently control both transparency/fluorescence intensity and fluorescence wavelength. From the perspective of fluorescent material design and processing, our approach offers a crucial advantage.

**Figure 5:**
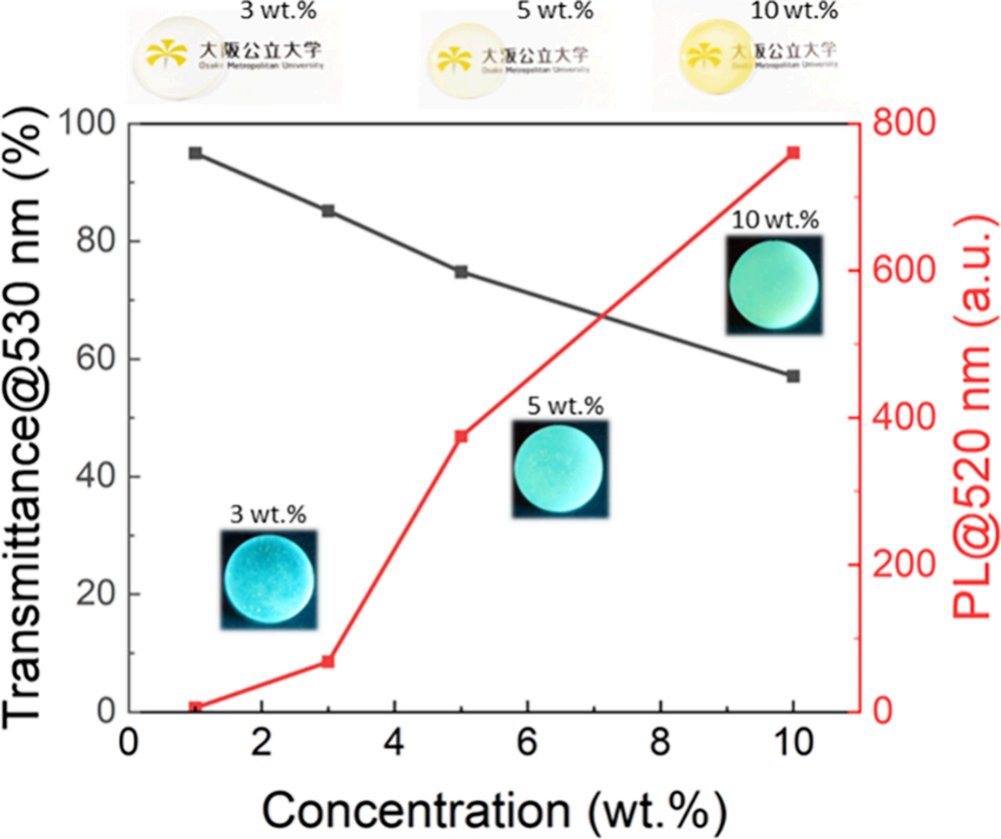
Optical transmittance (at 530 nm) and photoluminescence intensity (at 520 nm under excitation of 450 nm light) of the composite disks prepared by mixing C-dot-embedded SiO_2_ microspheres (6.0 × 10^–1^ wt % C-dot) and PMMA resin with varying concentrations.

Furthermore, the miscibility of these C-dot-embedded SiO_2_ microspheres with photocurable PMMA enables their facile shaping into various forms through simple molding or advanced stereolithography (Figure 6a). By blending the C-dot-embedded SiO_2_ microspheres with UV-curable acrylic resin and subsequently curing the mixture with 405 nm LED light after pouring it into a mold, a wide range of fluorescent objects can be formed. In these fluorescent objects, the emission wavelength can also be easily tuned from blue (1.0 × 10^–1^ wt % C-dots) to yellow (6.0 × 10^–1^ wt % C-dots) by simply adjusting the C-dot ratio in the precursor silica sol–gel solution. This versatility, stemming from the easy integration of our acrylic resin-based fluorophores with other materials, opens up avenues for a wide array of groundbreaking applications. As an illustrative example, Figure 6b demonstrates a chip where a prefabricated fluorescent object (as shown in Figure 6a), created via stereolithography, is embedded within an acrylic resin. One side (the front side) of this object is then coated with a blend of white ink and PMMA. As depicted in Figure 6b, this composite chip remains largely invisible under ambient white light, appearing as a plain white surface. However, upon irradiation with UV light, the prefabricated fluorescent object becomes distinctly visible, effectively ″emerging″ from the white background. This unique ″hidden-to-revealed″ functionality holds significant promise across a diverse range of applications. For instance, it offers advanced solutions for high-level security features and anticounterfeiting measures in sensitive documents, branded goods, and pharmaceuticals, where hidden patterns or information can be verified only under UV light. Beyond conventional static displays, this technology paves the way for innovative interactive signage and dynamic information displays, enabling hidden messages or advertisements to emerge only when illuminated by UV light, captivating audiences and creating novel discovery experiences. Furthermore, it unlocks creative possibilities in consumer products like modular assembly toys or creative building kits, allowing for the integration of hidden designs or interactive play elements that are revealed through UV light, fostering enhanced engagement and imagination. The ability to precisely control emission wavelength from blue to yellow by adjusting the C-dot concentration, combined with the versatility of an acrylic resin base, ensures the broad applicability and easy integration of these advanced fluorescent composites into various real-world scenarios.

**Figure 6:**
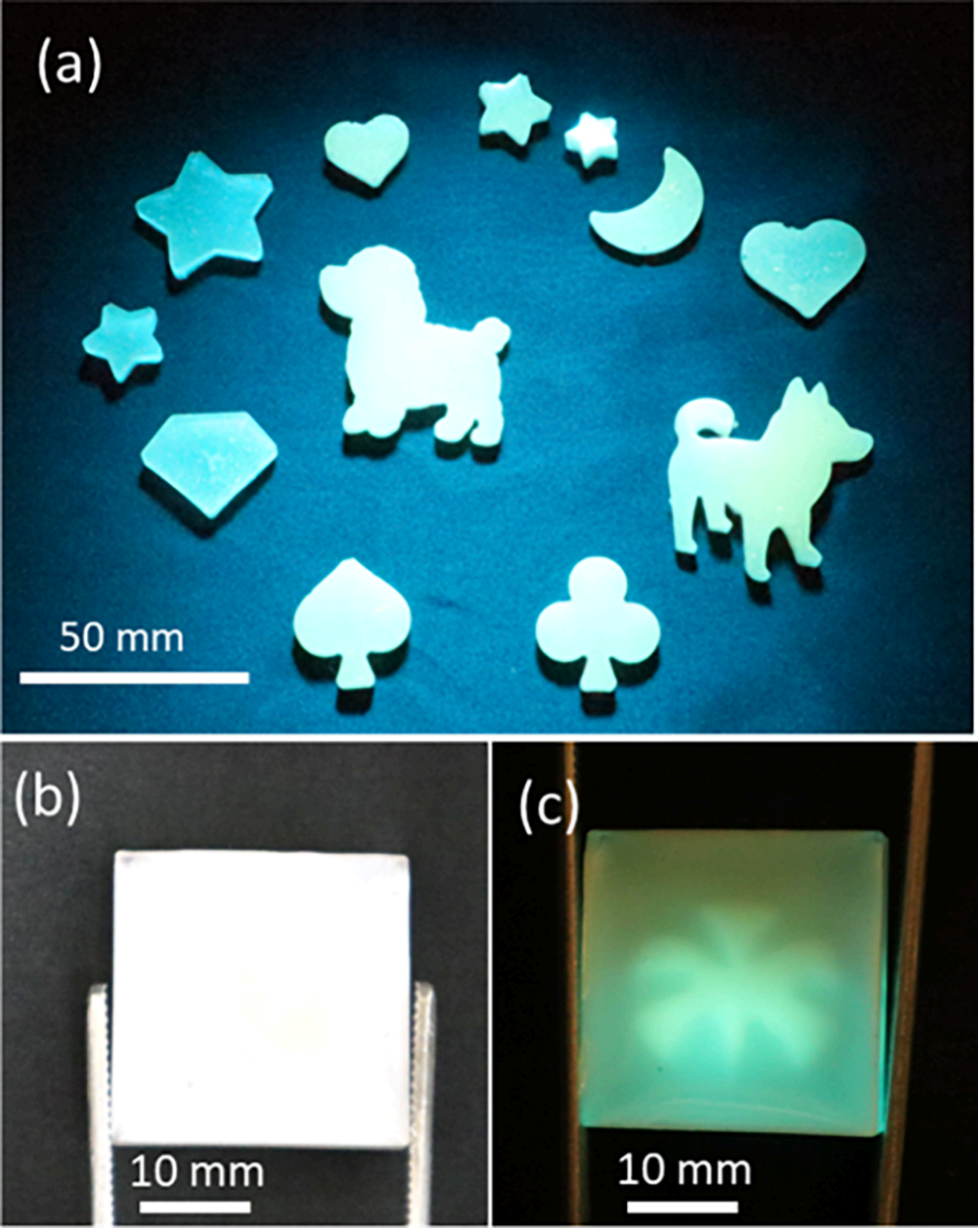
(a) Various fluorescent objects formed by blending C-dot-embedded SiO_2_ microspheres with UV-curable acrylic resin, demonstrating diverse shapes and tunable emission wavelengths (ranging from blue to yellow) under UV illumination. (b) A composite chip appearing white under ambient light due to its coated front side, and (c) the same chip under UV illumination (405 nm), revealing the hidden, prefabricated fluorescent object.

In industrial applications, injection molding is commonly used for shaping polymer-matrix fluorescent materials like PMMA. This process typically requires high temperatures, ranging from 150 to 350 °C, which are characteristic of the melting points of the polymers used. Consequently, fluorescent materials with poor heat resistance, such as C-dots, consistently suffer from thermal degradation and a decrease in fluorescence properties, rendering them unsuitable for injection molding processes. In contrast, the C-dot-embedded SiO_2_ microspheres synthesized in this study are expected to exhibit high thermal stability. This is attributed to the formation of a dense SiO_2_ layer on their surface as discussed above, which not only ensures robust retention of C-dots but also effectively hinders the access of oxygen gas to the interior of the particles.

To investigate this, we heated the C-dot-embedded SiO_2_ microspheres for 1 h at 50 °C intervals, ranging from 50 to 350 °C, encompassing typical injection molding temperatures. Subsequently, we measured their fluorescence intensity at 520 nm after heating the C-dot-embedded SiO_2_ microspheres at elevated temperature (Figure 7 and Figure S8). Remarkably, even after heating to 350 °C, the samples exhibited fluorescence intensities comparable to those of the as-synthesized samples, with no noticeable decrease in intensity. For comparison, a C-dot-containing silica bulk prepared by the sol–gel method underwent the same experiment. This comparative sample discolored and showed no fluorescence after just 5 min of heating at 350 °C. Considering the gas adsorption measurement results shown in Figure 3, it is highly probable that the dense surface silica layer in the present C-dot-embedded SiO_2_ microspheres suppressed the diffusion of oxygen gas into the particle interior, thereby contributing to a significant improvement in thermal resistance. Thus, this approach, employing the spray-drying method, successfully encapsulates C-dots within SiO_2_ microspheres, enhancing their thermal stability and enabling their application in high-temperature injection molding processes previously deemed unsuitable.^39–41^ We demonstrated this by incorporating C-dot-embedded SiO_2_ microspheres into PMMA, allowing for the formation of pellets through injection molding at 250 °C (Figure 8 and Figure S9). Furthermore, these pellets enabled the creation of C-dot phosphors in desired shapes via injection molding into molds at 250 °C. This breakthrough represents a significant advancement, as it effectively broadens the applicability of inherently thermally sensitive carbon dots to widely utilized industrial polymer processing techniques, opening new avenues for their large-scale production and integration into various functional materials.

**Figure 7:**
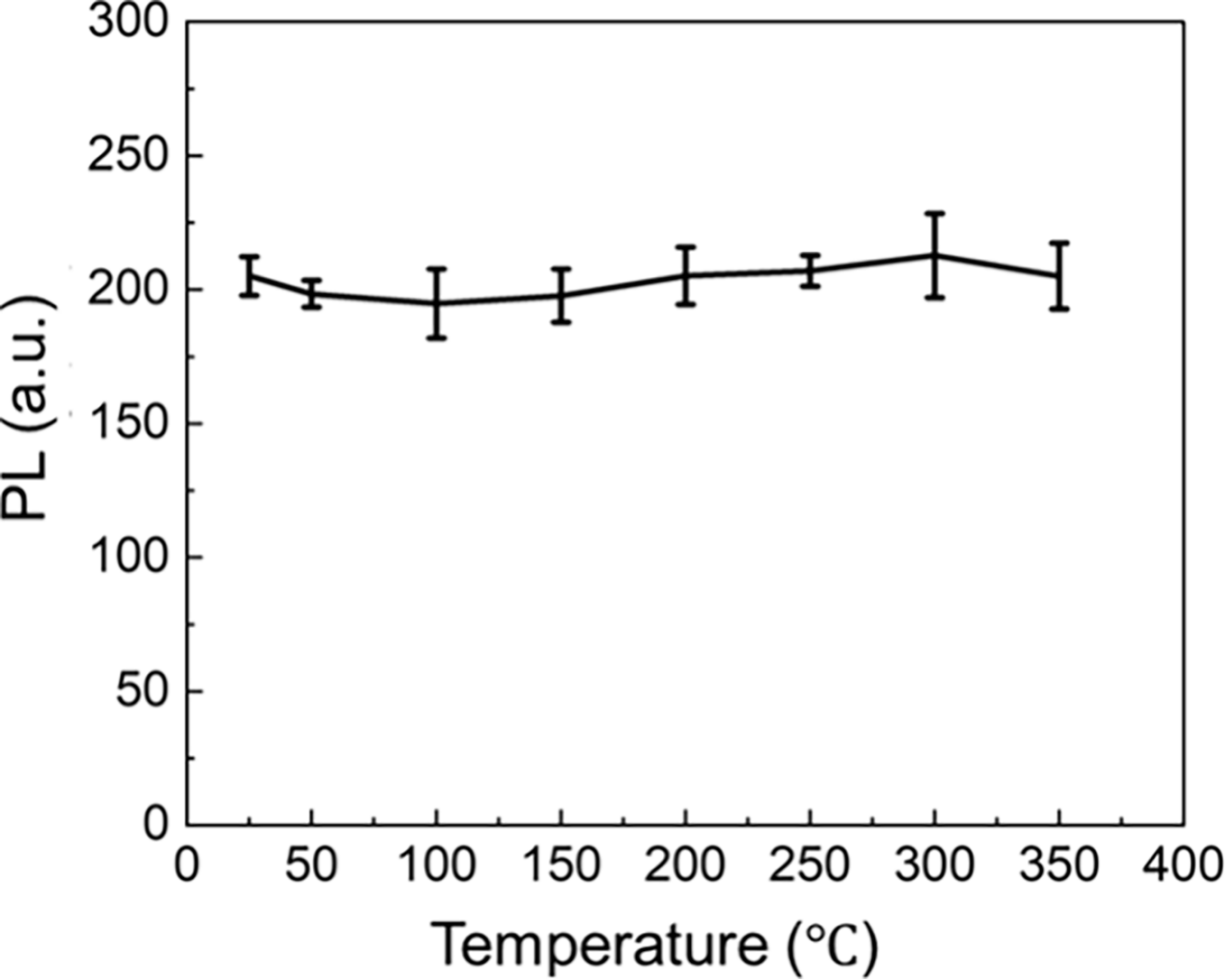
Photoluminescence intensity at 520 nm after heating the C-dot-embedded SiO_2_ microspheres at elevated temperatures for 1 h (excitation at 450 nm).

**Figure 8:**
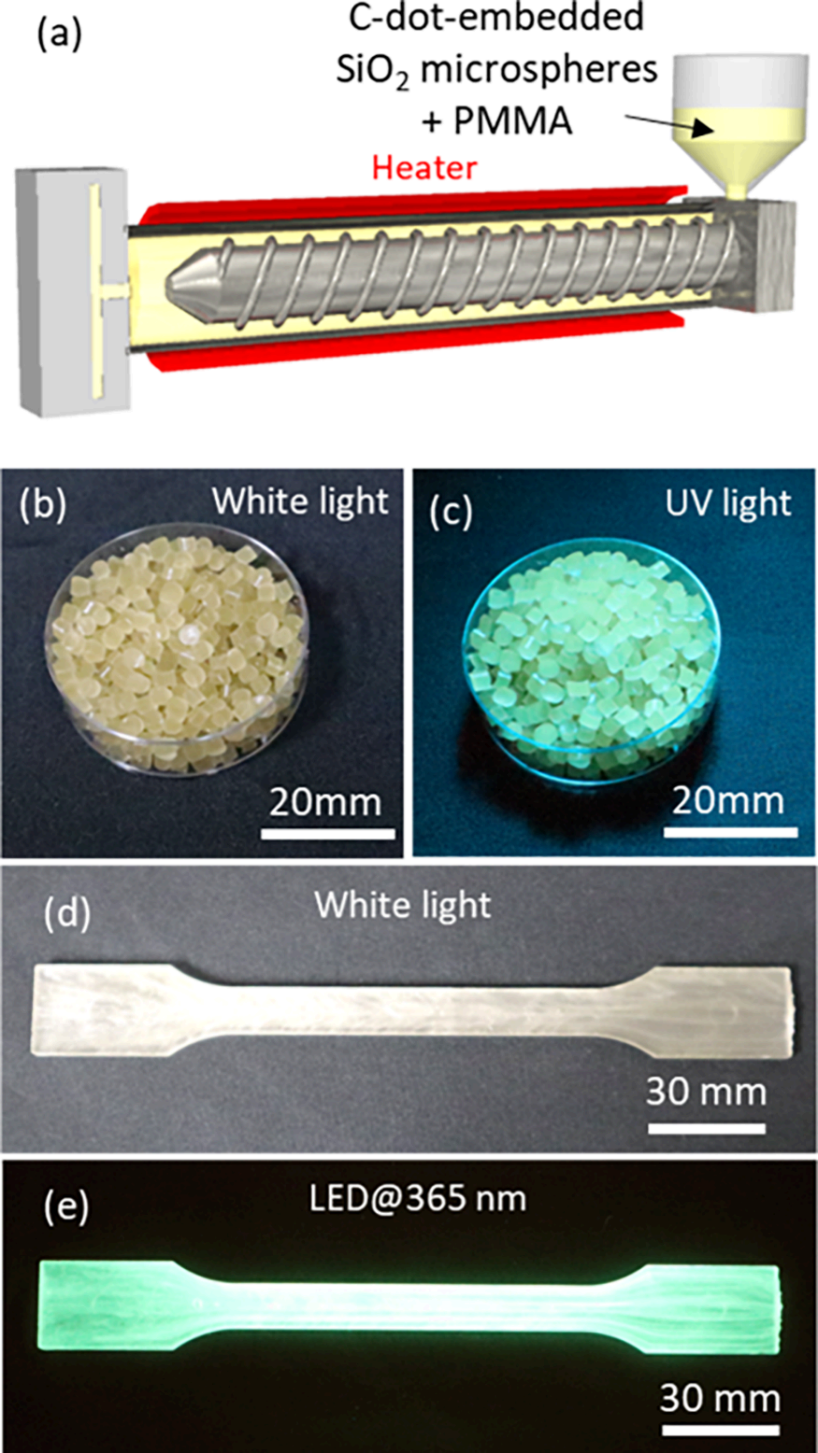
(a) A schematic illustration showing injection molding using a resin composed of C-dot-embedded SiO_2_ microspheres and PMMA. (b–e) Photos of pellets and a dumbbell-shaped object of C-dot phosphors prepared by the injection molding process.

## 4 Conclusions

In this study, we successfully synthesized C-dot-embedded SiO_2_ microspheres with a shell characterized by low accessible porosity using a spray-drying method. This novel structure provided a dense, nonporous silica shell that effectively protected C-dots from environmental degradation and, critically, significantly enhanced their thermal stability up to 350 °C. This allowed for the successful incorporation of C-dots into PMMA via high-temperature injection molding (250 °C), expanding their processability into complex shapes previously unattainable.

Furthermore, the C-dot-embedded SiO_2_ microspheres demonstrated unprecedented control over optical properties: the emission wavelength could be precisely tuned by varying C-dot concentration within the silica, while emission intensity and optical transparency could be independently controlled by adjusting the microsphere concentration in the PMMA matrix. This independent control, along with excellent long-term stability and compatibility with injection molding and stereolithography, offers substantial industrial advantages. Our findings pave the way for the broad application of C-dot-based fluorescent materials in fields such as large-area displays, flexible devices, and biocompatible medical applications.

## Supplementary Material



## Notes

XRD pattern, SEM images of the particles and excitation–emission intensity spectra of the composite disks with varying concentrations in detail (PDF)

## References

[1] Li H., Yan X., Kong D., Jin R., Sun C., Du D., Lin Y., Lu G. (2020). Recent advances in carbon dots for bioimaging applications. Nanoscale Horizons.

[2] Song Y., Zhu S., Yang B. (2014). Bioimaging
based on fluorescent carbon dots. RSC Adv..

[3] Lewis J. S., Weaver M. S. (2004). Thin-Film Permeation-Barrier Technology for Flexible
Organic Light-Emitting Devices. IEEE J. Sel.
Top. Quantum Electron..

[4] Meyer J., Görrn P., Bertram F., Hamwi S., Winkler T., Johannes H. H., Weimann T., Hinze P., Riedl T., Kowalsky W. (2009). Al2O3/ZrO2
Nanolaminates as Ultrahigh Gas-Diffusion
Barriers–A Strategy for Reliable Encapsulation of Organic Electronics. Adv. Mater..

[5] Kar D. K., V P., Si S., Panigrahi H., Mishra S. (2024). Carbon Dots and Their
Polymeric Nanocomposites: Insight into Their Synthesis, Photoluminescence
Mechanisms, and Recent Trends in Sensing Applications. ACS Omega.

[6] Malfatti L., Innocenzi P. (2018). Sol-Gel Chemistry
for Carbon Dots. Chem. Rec..

[7] Zhou Y., Qu Z. B., Zeng Y., Zhou T., Shi G. (2014). A novel composite
of graphene quantum dots and molecularly imprinted polymer for fluorescent
detection of paranitrophenol. Biosens. Bioelectron..

[8] Mehrzad-Samarin M., Faridbod F., Ganjali M. R. (2019). A luminescence
nanosensor for Ornidazole
detection using graphene quantum dots entrapped in silica molecular
imprinted polymer. Spectrochim. Acta A: Mol.
Biomol. Spectrosc..

[9] Nandy S., Kundu D., Naskar M. K. (2014). Synthesis of mesoporous
Stöber
silica nanoparticles: the effect of secondary and tertiary alkanolamines. J. Sol-Gel Sci. Technol..

[10] Gholami T., Salavati-Niasari M., Bazarganipour M., Noori E. (2013). Synthesis and characterization
of spherical silica nanoparticles by modified Stöber process
assisted by organic ligand. Superlattices Microstruct..

[11] Dasog M., Yang Z., Veinot J. G. C. (2012). Size-controlled
solid state synthesis
of luminescent silicon nanocrystals using Stöber silica particles. CrystEngComm.

[12] Zuo X., Xia Y., Ji Q., Gao X., Yin S., Wang M., Wang X., Qiu B., Wei A., Sun Z. (2017). Self-Templating Construction of 3D Hierarchical
Macro-/Mesoporous
Silicon from 0D Silica Nanoparticles. ACS Nano.

[13] Wang X. D., Shen Z. X., Sang T., Cheng X. B., Li M. F., Chen L. Y., Wang Z. S. (2010). Preparation of spherical silica particles
by Stober process with high concentration of tetra-ethyl-orthosilicate. J. Colloid Interface Sci..

[14] Takeuchi H., Nagira S., Yamamoto H., Kawashima Y. (2004). Solid dispersion
particles of tolbutamide prepared with fine silica particles by the
spray-drying method. Powder Technol..

[15] Cai Y. Z., Corke H. (2000). Production
and Properties of Spray-dried Amaranthus Betacyanin Pigments. J. Food Sci..

[16] Kim C. S., Ahn K. W., Rah S. C., Kim S.-G. (2008). Preparation of Silica
Nanostructured Spheres by Sol Spray Drying. Drying Technol..

[17] Li M., Hou X., Sha Y., Wang J., Hu S., Liu X., Shao Z. (2014). Facile spray-drying/pyrolysis synthesis of core–shell
structure
graphite/silicon-porous carbon composite as a superior anode for Li-ion
batteries. J. Power Sources.

[18] Pitchumani R., Heiszwolf J. J., Schmidt-Ott A., Coppens M. O. (2009). Continuous synthesis
by spray drying of highly stable mesoporous silica and silica–alumina
catalysts using industrial raw materials. Microporous
Mesoporous Mater..

[19] Kairdolf B. A., Smith A. M., Stokes T. H., Wang M. D., Young A. N., Nie S. (2013). Semiconductor quantum
dots for bioimaging and biodiagnostic applications. Annu. Rev. Anal. Chem..

[20] Deng D., Chen Y., Cao J., Tian J., Qian Z., Achilefu S., Gu Y. (2012). High-Quality
CuInS2/ZnS Quantum Dots
for In vitro and In vivo Bioimaging. Chem. Mater..

[21] Lu H., Li W., Dong H., Wei M. (2019). Graphene Quantum Dots for Optical
Bioimaging. Small.

[22] Biswas M. C., Islam M. T., Nandy P. K., Hossain M. M. (2021). Graphene
Quantum
Dots (GQDs) for Bioimaging and Drug Delivery Applications: A Review. ACS Mater. Lett..

[23] Wang Y., Hu R., Lin G., Roy I., Yong K. T. (2013). Functionalized quantum
dots for biosensing and bioimaging and concerns on toxicity. ACS Appl. Mater. Interfaces.

[24] Ding C., Zhu A., Tian Y. (2014). Functional
surface engineering of C-dots for fluorescent
biosensing and in vivo bioimaging. Acc. Chem.
Res..

[25] Luo P. G., Sahu S., Yang S. T., Sonkar S. K., Wang J., Wang H., LeCroy G. E., Cao L., Sun Y. P. (2013). Carbon
″quantum″ dots for optical bioimaging. J. Mater. Chem. B.

[26] Zhao Y. B., Ma Y. J., Song D., Liu Y., Luo Y., Lin S., Liu C. Y. (2017). New luminescent
nanoparticles based on carbon dots/SiO_2_ for the detection
of latent fingermarks. Anal. Methods.

[27] Wintzheimer S., Luthardt L., Cao K. L. A., Imaz I., Maspoch D., Ogi T., Bück A., Debecker D. P., Faustini M., Mandel K. (2023). Multifunctional,
Hybrid Materials Design via Spray-Drying: Much more than Just Drying. Adv. Mater..

[28] Iskandar F., Mikrajuddin, Okuyama K. (2002). Controllability of Pore Size and Porosity on Self-Organized
Porous Silica Particles. Nano Lett..

[29] Iskandar F., Gradon L., Okuyama K. (2003). Control of
the morphology of nanostructured
particles prepared by the spray drying of a nanoparticle sol. J. Colloid Interface Sci..

[30] Dos
Santos da Silva A., Dos Santos J. H. Z. (2023). Stober method and its nuances over
the years. Adv. Colloid Interface Sci..

[31] Iwabayashi H., Okada K., Fukatsu A., Suzuki K., Mori R., Takahashi M. (2025). Carbon Dot-Doped
Silica Xerogel Phosphors Excited by
Blue LEDs and LDs for the Brilliant White Lighting of Endoscope Tips. Adv. Mater. Interfaces.

[32] Tokudome Y., Kuniwaki H., Suzuki K., Carboni D., Poologasundarampillai G., Takahashi M. (2016). Thermoresponsive
Wrinkles on Hydrogels for Soft Actuators. Adv.
Mater. Interfaces.

[33] Takahashi M., Maeda T., Uemura K., Yao J., Tokuda Y., Yoko T., Kaji H., Marcelli A., Innocenzi P. (2007). Photoinduced
Formation of Wrinkled Microstructures with Long-Range Order in Thin
Oxide Films. Adv. Mater..

[34] Tokudome Y., Suzuki K., Kitanaga T., Takahashi M. (2012). Hierarchical
nested wrinkles on silica-polymer hybrid films: stimuli-responsive
micro periodic surface architectures. Sci. Rep..

[35] Hu Y., Hao D., Gong F., Gao Y., Yan X., Ma G. (2021). Facile one-pot
emulsion/sol-gel method for preparing wrinkled silica microspheres. Particuology.

[36] Yuan H., Wu K., Zhang J., Wang Y., Liu G., Sun J. (2019). Curvature-Controlled
Wrinkling Surfaces for Friction. Adv. Mater..

[37] Isnaeni, Herbani Y., Suliyanti M. M. (2018). Concentration
effect on optical properties of carbon dots at room temperature. J. Lumin..

[38] Feng Z., Adolfsson K. H., Xu Y., Fang H., Hakkarainen M., Wu M. (2021). Carbon dot/polymer nanocomposites:
From green synthesis to energy,
environmental and biomedical applications. Sustain.
Mater. Technol..

[39] Hernández-del-Valle M., Ilarraza-Zuazo J., Dios-Lázaro E., Rubio J., Audoux J., Haranczyk M. (2024). Pellet dispensomixer
and pellet distributor: open hardware
for nanocomposite space exploration via automated material compounding. Digit. Discovery.

[40] Schäfer C., Meyer S. P., Osswald T. A. (2018). A novel
extrusion process for the
production of polymer micropellets. Polym. Eng.
Sci..

[41] Besco S., Modesti M., Lorenzetti A. (2013). Influence of processing parameters
on the structure of melt blended polyethylene/organoclay nanocomposites
produced by a masterbatch route. Polym. Eng.
Sci..

