# Supplementary material for: Scalable Synthesis of Thermally Robust Carbon Dot-Silica Microspheres Enabling High-Temperature Polymer Processing and Multifunctional Luminescent Composites
Source: ACS Appl Mater Interfaces. 2026 Jan 8;18(2):4370–7. doi: 10.1021/acsami.5c22439 (PMC12828719; doi:10.1021/acsami.5c22439)
Supplement: Supplementary file 1 [file am5c22439_si_001.pdf]

Supporting Information

Scalable Synthesis of Thermally Robust Carbon Dot-Silica Microspheres Enabling High-Temperature Polymer Processing and Multifunctional Luminescent Composites

Hirohisa Iwabayashi, Kenji Okada, Arisa Fukatsu, Ryohei Mori, Masahide Takahashi**

H. Iwabayashi, K. Okada, A. Fukatsu, M. Takahashi
Department of Materials Science, Graduate School of Engineering, Osaka Metropolitan University
Naka-ku, Sakai
Osaka 599-8531, Japan
E-mail: k_okada@omu.ac.jp, masa@omu.ac.jp

H. Iwabayashi, R. Mori
Research Division, Fuji Pigment Co., Ltd.
Obana, Kawanishi
Hyogo 666-0015, Japan

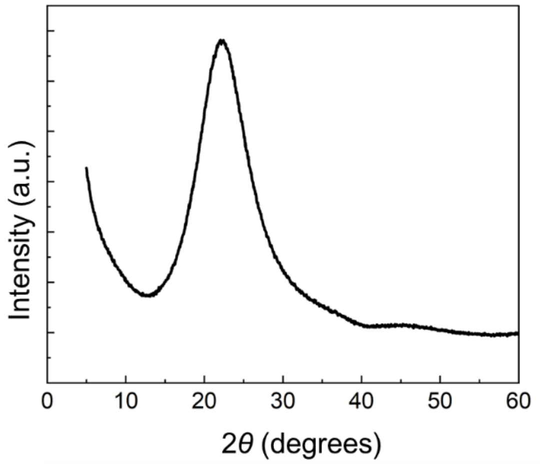

Figure S1. XRD pattern of the C-dot-embedded SiO_2 microspheres (6.0×10^{-1} wt.% C-dot).

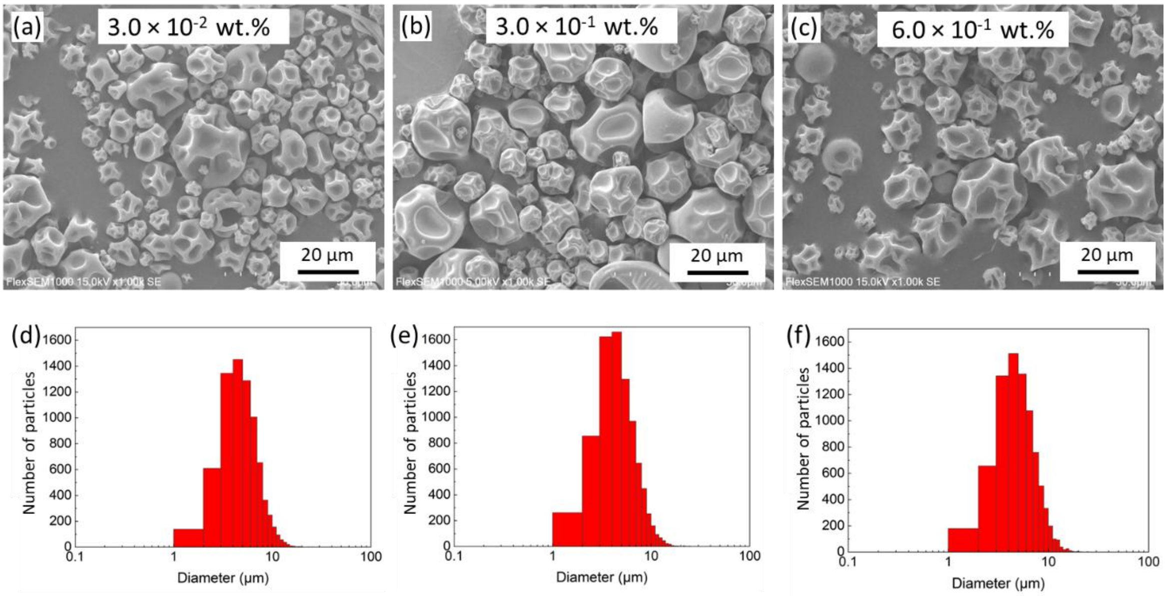

Figure S2. SEM images and their particle size distributions of the C-dot-embedded SiO_2 microspheres prepared from the silica sol-gel solution with varying C-dot concentrations ((a, d) 3.0×10^{-2} wt.%, (b, e) 3.0×10^{-1} wt.% and (c, f) 6.0×10^{-1} wt.%).

Table S1. Average particle size (D10, D50, and D90) and the total number of particles counted for C-dot-embedded SiO_2 microspheres.

**C-dot content (wt%)**	**D10 (μm)**	**D50 (μm)**	**D90 (μm)**	**Particles Counted**
0.03	2.97	5.09	8.59	7500
0.3	2.75	4.83	8.51	8438
0.6	2.95	5.26	8.94	8223

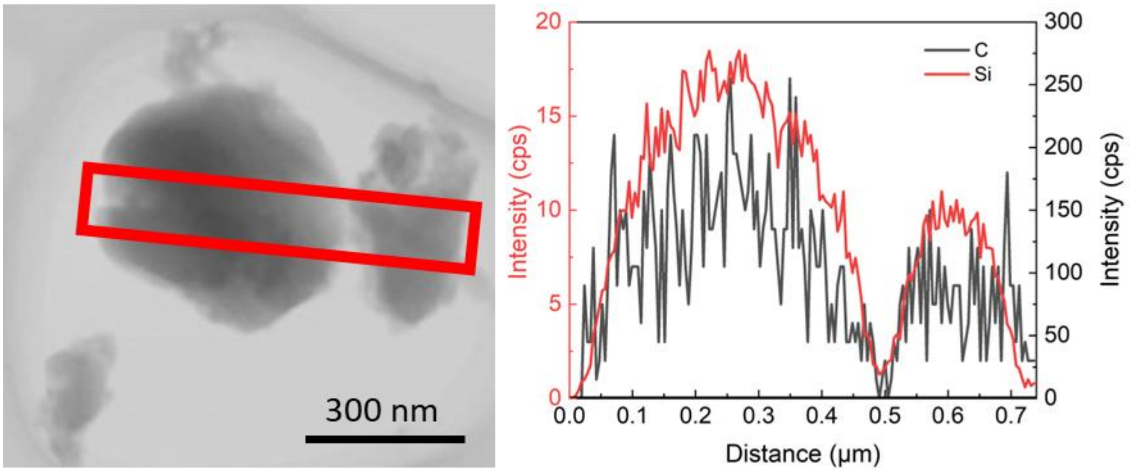

Figure S3. STEM image and corresponding EDS elemental profiles of a fractured SiO_2 microsphere fragment. The profiles were obtained by integrating the signal intensity within the rectangular area (red box) to improve the signal-to-noise ratio. The co-localization of Si (red) and C (black) signals confirms the uniform distribution of C-dots within the silica matrix.

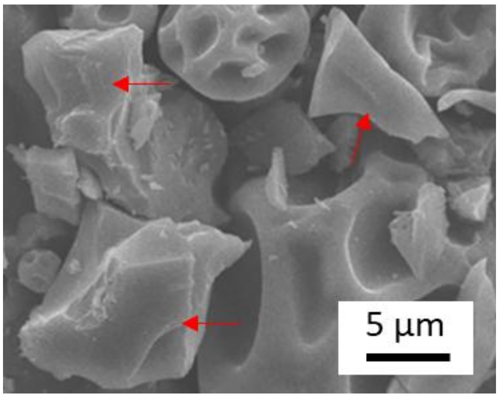

Figure S4. SEM image of the cross-sections of the fractured C-dot-embedded SiO_2 microspheres as indicated by red arrows.

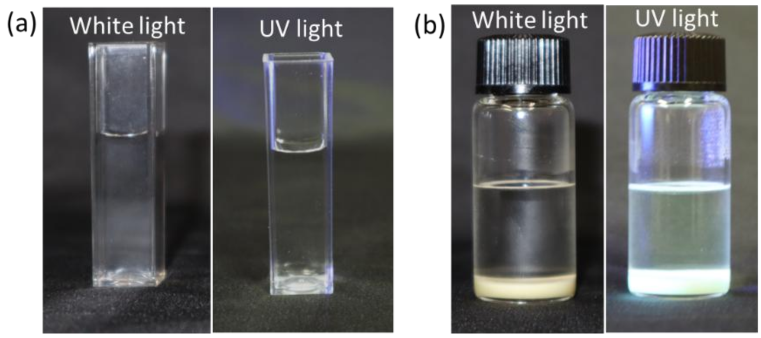

Figure S5. (a) Photos of supernatant solution after the C-dot-embedded SiO_2 microspheres were dispersed in deionized water, stirred, and then allowed to stand for one week. While, the precipitation (b) in the deionized water showed photoluminescence even after one week.

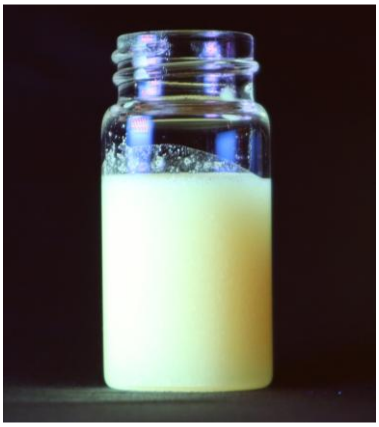

Figure S6. C-dot-embedded SiO_2 microspheres were homogeneous dispersed in PMMA resin. The photo was taken under UV light (365 nm).

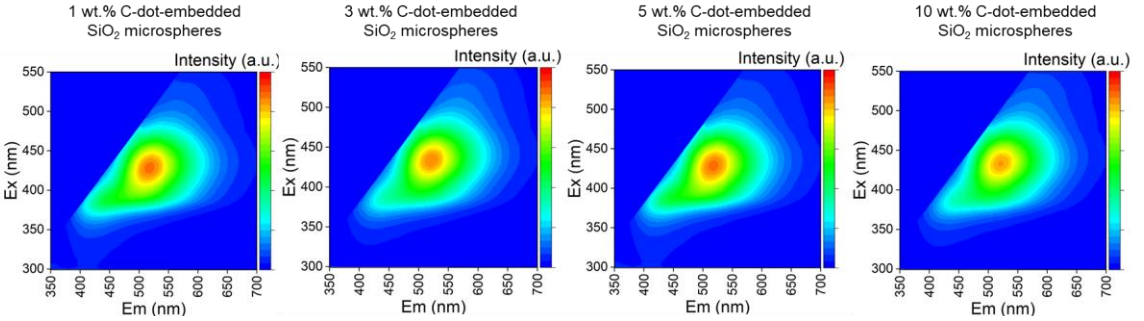

Figure S7. Excitation-emission intensity spectra of the composite disks prepared by mixing C-dot-embedded SiO_2 microspheres (6.0×10^{-1} wt.% C-dot) and PMMA resin with varying concentrations.

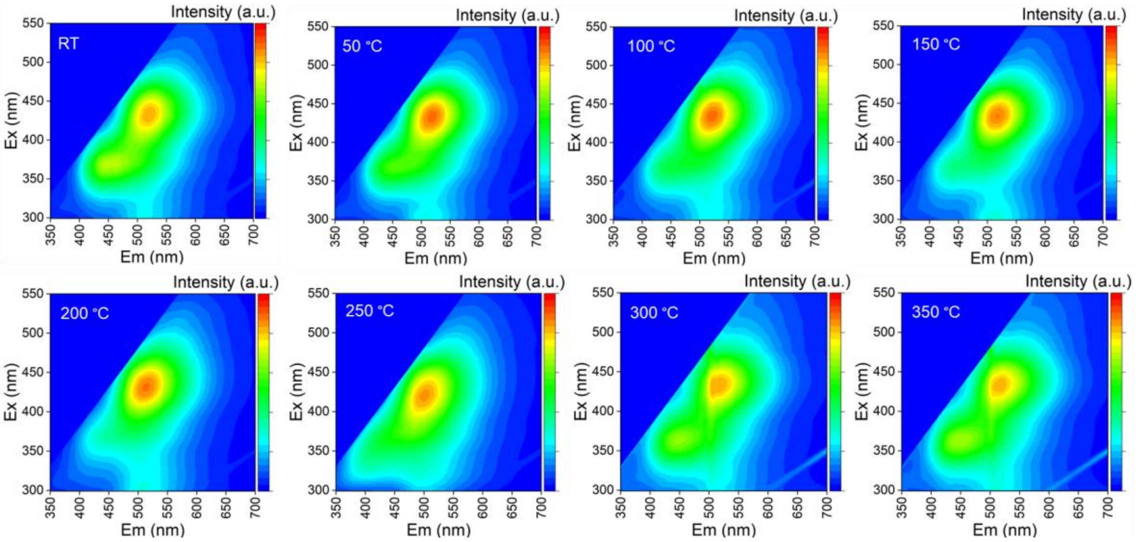

Figure S8. Excitation-emission (Ex-Em) contour maps of C-dot-embedded SiO_2 microspheres after heating for 1 hour at various temperatures (from room temperature (RT) to 350 °C), demonstrating their excellent thermal stability.

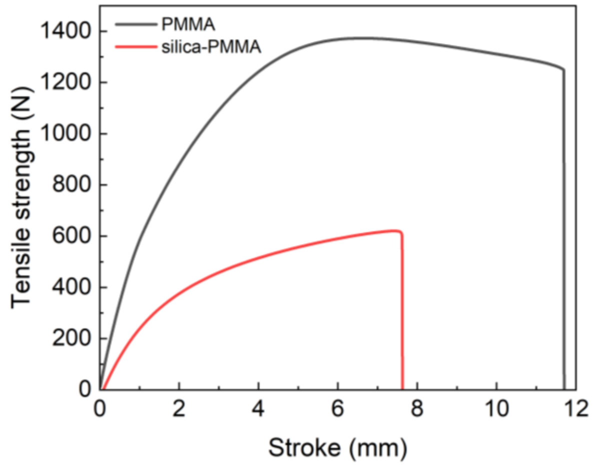

Figure S9. Representative stress-strain curves of neat PMMA and the C-dot-embedded SiO_2 /PMMA composite.
